# Supplementary material for: Content-rich biological network constructed by mining PubMed abstracts
Source: BMC Bioinformatics. 2004 Oct 8;5:147. doi: 10.1186/1471-2105-5-147 (PMC528731; doi:10.1186/1471-2105-5-147)
Supplement: Additional File 5 — The original Chilibot query results of the term "long-term potentiation (LTP)" and 22 other terms, limiting the latest references analyzed to the years 1990, 1995, 2000, and 2004. [file 1471-2105-5-147-S5.bz2 › chilibotAdditionalFile5/ltp2004/html/PI-3K_AMPA.html]

 


 **PI-3K** and **AMPA** 
  
Found 4 abstracts in PubMed,  **4 abstracts were retrieved and analyzed**.  


---

 Search Google  |
 PDF files only 
|  EDU domain only 

---

**Interactive relationship** (e.g. stimulation, inhibition, etc)

- We previously demonstrated that calcium permeable  **AMPA**  receptors activate Erkl 2 through a phosphatidylinositol 3 kinase PI 3 kinase  [ **PI-3K** ]  dependent mechanism.  Ref: 11902114 J Neurochem, 2002
- Our results indicate that calcium permeable  **AMPA**  receptors transduce signals from the cell surface to the nucleus of neurons through a PI 3 kinase  [ **PI-3K** ]  dependent activation of MAPK.  Ref: 10407026 J Neurosci, 1999
- However,  **AMPA**  receptor stimulation led to a calcium dependent phosphorylation of the nuclear transcription factor CREB, which could be prevented by inhibitors of MEK or PI 3 kinase  [ **PI-3K** ] .  Ref: 10407026 J Neurosci, 1999
- Activation of PI3 kinase  [ **PI-3K** ]  is required for  **AMPA**  receptor insertion during LTP of mEPSCs in cultured hippocampal neurons.  Ref: 12765612 Neuron, 2003
